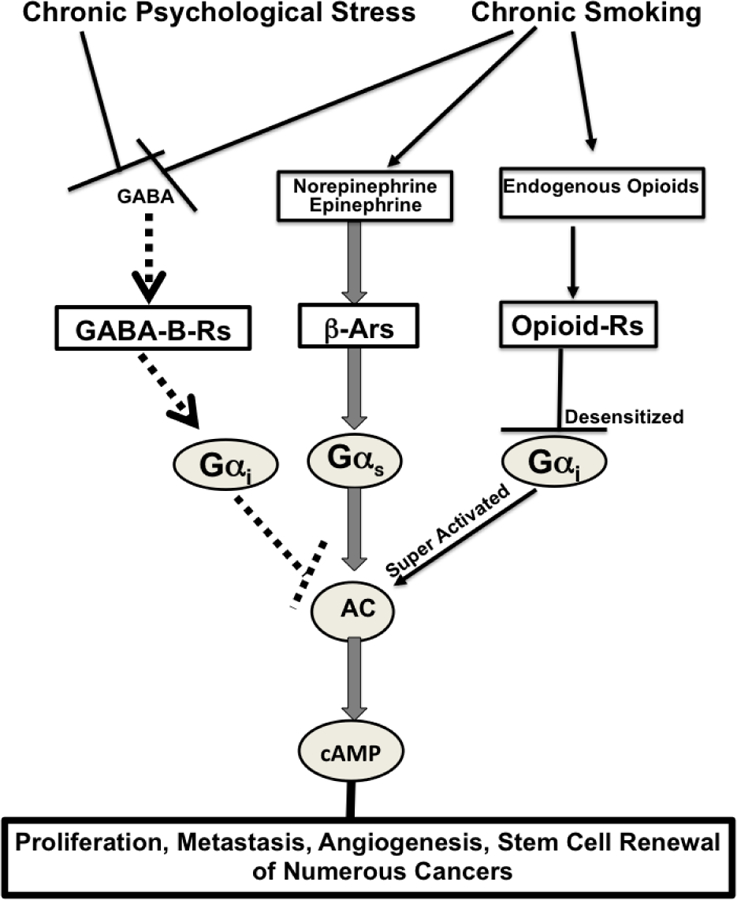# The Neuro-Psychological Axis of Smoking-Associated Cancer

**DOI:** 10.29245/2578-3009/2019/2.1166

**Published:** 2019-03-19

**Authors:** Hildegard M Schuller

**Affiliations:** Department of Biomedical & Diagnostic Sciences, College of Veterinary Medicine, University of Tennessee, 2407 River Drive, Knoxville, TN 37996, USA

## Abstract

This mini-review summarizes current knowledge on similarities and synergism between smoking and psychological stress-induced modulations of growth stimulating and inhibiting regulatory networks in epithelial cells and epithelial cancers with emphasis on cancer stimulating neurotransmitters and their receptors as well as cancer inhibiting γ-aminobutyric acid (GABA) and opioids. Hyperactive cAMP signaling downstream of beta-adrenergic receptors (β-ARs) has been identified as the driving force of most smoking-associated cancers by numerous preclinical studies and psychological stress intensifies these effects while experimental stress reduction inhibits. The integration of cAMP reduction via stress reduction by pharmacological and psychological means such as psychotherapy, relaxation meditation and yoga into any cancer treatment strategy is recommended.

## Introduction

Smoking is a documented risk factor for numerous human cancers, including cancer of the lungs, larynx, esophagus, stomach, breast, pancreas, colon, prostate and bladder^1–3^, with a particularly strong etiological association between smoking and cancer of the larynx, lungs^4^ and pancreas^5^. Research into the mechanisms of tobacco-associated carcinogenesis has identified several powerful carcinogens in tobacco smoke, including polycyclic aromatic hydrocarbons (predominantly benzo[a]pyrene) and the nicotine derived nitrosamines N’-nitrosonornicotine (NNN) and 4-(methylnitrosamino)-1-(3-pyridyl)-1-butanone (N’nitrosonicotine ketone, NNK)^1^.

Research into the mechanisms of action of tobacco carcinogens has identified interactions of their metabolites with DNA that result in the formation of inactivating mutations in the tumor suppressor gene *p53*^*6*^ and in mutations in the *k-ras* gene^6^ that sensitize the gene to its upstream stimulators^7^. Both mutations are frequently expressed in tobacco-associated human cancers^8–9^. Moreover, NNN and NNK are agonists for nicotinic acetylcholine receptors (nAChRs) with significantly higher affinity than their physiological agonist acetylcholine or nicotine^10^ and NNK is additionally an agonist for beta-adrenergic receptors (β-ARs) with significantly higher affinity than their physiological agonists epinephrine (Epi) and norepinephrine (Nor)^11^. In light of the ubiquitous expression of nAChRs and β-ARs in mammalian cells^12–13^, these findings prompted research into the potential role of neurotransmitter receptors of the nicotinic cholinergic and beta-adrenergic families in the development, progression and resistance to therapy of cancer.

## Regulation of cancer by the nAChR-mediated release of neurotransmitters.

The excitatory neurotransmitters acetylcholine, serotonin, glutamate, dopamine, Epi and Nor are not only synthesized and released by the brain and the autonomic nervous system but also by normal epithelial cells and epithelial cancers^12^, ^14–17^ and Nor and Epi in addition by the adrenal gland^18^. Acetylcholine is the physiological agonist for nAChRs and opens their ion channel upon binding to the receptor, resulting in membrane depolarization that triggers the opening of voltage-gated Ca^2+-^channels. In turn, this allows for the influx of Ca^2+^ ions, causing the release of neurotransmitters^19^.

Early *in vitro* studies have shown that binding of nicotine to the α7nAChR regulates the autocrine regulation of cell proliferation by serotonin in small cell lung cancer cells^20^. In addition, it has been shown that increases in systemic serotonin stimulated the growth of colon cancer allografts in mice by inducing angiogenesis^21^.

More recent investigations have shown that the α7nAChR regulates the release of Epi and Nor *in vitro* from cells of normal small airway epithelium, lung adenocarcinoma^16^, pancreatic duct epithelia and pancreatic ductal adenocarcinoma^15^, gastric cancer^22^, colon cancer^23^, and urothelial bladder cancer^24^ and induces their proliferation and migration via this autocrine mechanism. Moreover, all of these cancers as well as prostate cancer, ovarian cancer, breast cancer, and hemangiosarcoma are stimulated in their growth by exposure to exogenous Epi, Nor or synthetic beta-adrenergic agonist while the non-selective beta-blocker propranolol inhibits the autocrine and exogenous stimulation of these cancers^24–26^. In addition, it has been shown in adenocarcinomas of the lung and pancreas that NNK has identical cancer-stimulating effects as Epi and Nor by binding as an agonist to β-ARs and that propranolol inhibited these responses^11, 25^.

The amino acid neurotransmitter glutamate is synthesized and released by numerous cancers and stimulates their proliferation and migration, including cancer of the pancreas, prostate, breast and adenocarcinoma of the lungs^26^. In turn, the release of glutamate is regulated by the α7nAChR^27^. Moreover, the α7nAChR regulates the release of the catecholamine neurotransmitter dopamine and its receptors that are expressed in many cancers and can have cancer stimulating as well as inhibitory effects pending on the expression levels of receptors of the D1-like family which increase cAMP signaling via the G-protein G_sq_ or receptors of the D2-like family that are coupled to the inhibitory G-protein G_i_ and inhibit cAMP formation ^28–29^.

## Antagonistic effects of receptors coupled to the stimulatory G protein G_s_ and receptors coupled to the inhibitory G protein G_i_

Beta-adrenergic receptors are coupled to the stimulatory G protein G_s_. Activation of G_s_ by binding of an agonist to the receptor activates the enzyme adenylyl cyclase that catalyzes the formation of intracellular cyclic adenosine monophosphate (cAMP) which in turn activates protein kinase A (PKA)^30^. Increased intracellular cAMP and activated PKA stimulate the release of epidermal growth factor (EGF)^25, 31^, vascular endothelial growth factor (VEGF)^32–33^ and arachidonic acid (AA)^11^ from the cancer cells and from fibroblasts, macrophages and endothelial cells in the stroma that constitutes the cancer micro-environment^34–35^. Each one of these released products stimulates the growth, metastatic potential and resistance to therapy of cancer.

Receptors coupled to the inhibitory G protein G_i_ and their endogenous agonists are the physiological inhibitors of G_s_-coupled receptors. In accord with this function, it has been shown that the inhibitory neurotransmitter γ-aminobutyric acid (GABA) has tumor suppressor function via G_i_-coupled GABAB receptors in vitro and in animal models for adenocarcinoma of the lung^36^and pancreas^37^. Cancer stem cells isolated from pancreatic cancer cell lines stimulated their self-renewal by the autocrine release of Epi and Nor that activated beta-adrenergic signaling and these effects were blocked by treatment with GABA^38^. In addition, the opioid dynorphin B inhibited the self-renewal of cancer stem cells from lung adenocarcinomas via their G_i_-coupled opioid receptors^39^. Similarly, the synthetic opioid methadone has strong inhibiting effects on numerous cancers^40–41^. Moreover, preclinical studies have shown that the endogenous cannabinoid system is activated by binding of exogenous cannabinoids (medical marihuana, synthetic cannabinoids) to G_i_-coupled cannabinoid receptors, resulting in growth inhibition and improved response to therapy of lung adenocarcinoma, colon cancer and glioblastoma^42–44^.

## Effects of smoking and chronic psychological stress on cancer stimulating and inhibiting networks (Figure 1)

Smoking and chronic psychological stress each induce the nAChR-regulated release of Epi and Nor, thereby increasing their systemic levels^45^. In turn, this creates an environment that supports the development and progression of numerous cancers for which Epi and Nor act as strong growth factors. Smoking and chronic psychological stress additionally suppress the GABA system^46–47^, thus depriving the body of the physiological inhibitor of Epi and Nor-induced cancer stimulation. Acute exposure to nicotine stimulates the nAChR-mediated release of endogenous opioids above physiological levels^48^. Similar to opioid addiction, the continued exposure to unphysiologically high opioid levels during chronic nicotine-induced nicotine addiction and withdrawal desensitizes the G_i_-coupled opioid receptors^49^, resulting in a reactive super activation of adenylyl cyclase/cAMP signaling^48^, ^50^. On the other hand, stress reduction^51^ and positive emotions^52^ decrease the levels of stress neurotransmitters while simultaneously increasing the levels of GABA and endogenous opioids within their phsyiologal range, thereby restoring cAMP homeostasis. The strong influence of these neuropsychological factors on cancer development and progression has been documented by preclinical investigations which have reported significant cancer-stimulating effects of experimentally induced stress on cancer of the lungs^53^, pancreas^37^, breast^54–55^ and ovary^56^ whereas stress reduction by species appropriate environmental enrichment significantly reduced the development and progression of mouse xenografts from lung adenocarcinomas^39^.

## Conclusions and future directions

The addictive properties of smoking have been extensively investigated. The focus of that research has been on nicotine-induced changes in nAChR-mediated brain neurotransmission characterized by hyperactivity of excitatory neurotransmitters accompanied by suppression of their physiological inhibitors, the GABA and endogenous opioid systems, and the resulting psychological responses associated with addiction and withdrawal symptoms. However, the fact that smoking also causes cardiovascular disease by elevating systemic Epi and Nor levels due to their increased release from the adrenal gland and sympathetic nervous system strongly suggests that smoking-induced modulations in nAChR expression and function are not restricted to the brain, where they cause addiction, but instead occur universally in non-neuronal cells and tissues as well where their altered functions cause non-neuronal diseases. As is summarized in this mini-review, epithelial cells express nAChR-regulated autocrine signal transduction pathways that maintain the balance between excitatory neurotransmitters that stimulate cell proliferation and GABA which inhibits. The same changes that cause nicotine addiction when occurring in brain nAChRs cause systemic and epithelial hyperactivity of cancer stimulating neurotransmitters while suppressing inhibitory GABA. The release of Epi and Nor from cancer cells and the sympathetic nervous system is predominantly stimulated by the homomeric α7nAChR^14^ which does not undergo long-lasting desensitization in response to chronic nicotine exposure^57^. By contrast, the heteromeric α4β2nAChRs that regulates GABA release from epithelial cells and epithelial cancers desensitizes in response to chronic nicotine, resulting in suppressed GABA release^58^. The unrestricted growth of cancer cells is further supported by the systemic increase in Nor and Epi and simultaneous suppression of the endogenous opioid system and GABA system caused by smoking and chronic psychological stress. The resulting beta-adrenergic receptor hyperactivity additionally impairs the immune system,via cyclooxynenase-2-mediated suppression of CD8+ T cell responses^59^, an effect caused by the beta-adrenergic stimulation of arachidonic acid release in cancer cells^11^.

Current therapeutic strategies of cancer therapy aim to destroy existing cancer cells by chemotherapy, radiation and immunotherapy. These treatments shrink existing tumors, thereby often rendering them surgically resectable, resulting in significant increases in overall survival times. However, they do not remove the imbalance in cancer stimulating and inhibiting regulatory networks characterized by hyperactive cAMP signaling that is caused by smoking and chronic psychological stress, which often work synergistically. Accordingly, the majority of cancers eventually relapse. A major goal of adjuvant cancer therapy aimed at preventing the formation of new cancer cells via self-renewing stem cells and the associated progression, resistance to therapy and cancer relapse should therefore be the restoration of cAMP homeostasis. General beta-blockers such as propranolol used successfully for the long-term management of cardiovascular disease, nutritional GABA supplements or over the counter valerian extracts that stimulates the endogenous synthesis of GABA (both widely used as sleep aids and anxyolytics), positive allosteric modulators of the GABA_B_-R used for the management of drug addiction as well as opioids used for anesthesia, analgesia, cough suppression and the management of drug addiction should all be explored in clinical trials as adjuvant therapy of cancer. Finally, the powerful cancer stimulating effects of psychological stress and cancer inhibiting effects of psychological well –being cannot be over-emphasized. Stress reduction/relaxation by psychotherapy, relaxation meditation and yoga should be an essential component of any cancer treatment plan.

## Figures and Tables

**Figure 1:**